# Conducting a randomized controlled clinical trial on palliative care in patients with glioblastoma – what are the challenges?

**DOI:** 10.1007/s00520-026-10564-7

**Published:** 2026-04-27

**Authors:** Melanie Joshi, Charlotte Nettekoven, Sophia Kochs, Iris Appelmann, Claudia Bausewein, Gerhild Becker, Christopher Boehlke, Tzvetina Brumbarova, Daniele Civello, Hans Clusmann, Roland Goldbrunner, Birgit Haberland, Dieter Henrik Heiland, Martin Hellmich, Ulrich Herrlinger, Birgit Jaspers, Dirk Müller, Wiebke Müller, Chuh-Hyoun Na, Martin Neukirchen, Lukas Radbruch, Marion Rapp, Roman Rolke, Maximilian I. Ruge, Michael Sabel, Oliver Schnell, Jacqueline Schwartz, Niklas Thon, Hartmut Vatter, Louisa von Baumgarten, Raymond Voltz, Heidrun Golla, EPCOG Study Group, Manuela Langheimer, Marita Kumschlies, Daniel Delev, Norbert Krumm, Sonja Hiddemann, Hilde Cavelius, Imke Bronger, Christina Thamm, Christiane Landwehr, Mirco Muscheid, Erdem Güresir, Christina Schaub, Niklas Schäfer, Michaela Hesse, Berit Zimmer, Kirsten Hüning, Claudia Stratmann, Derya Tezel, Caterina Quente, Natalie Meyer-Sevens, Nicole Dietrichs, Lena Koschnitzke, Marcel Kamp, Marie Schulz, Petra Winter, Manuela Schallenburger, Nadja Jarc, Nicole Koch, Anja Wiegleb-Brunet, Yashar Naseri, Bianca Blaß, Marcia Machein, Nicolas Neidert, Pamela Heiland, Debora Cipriani, Anna Vongerichten, Mateo Tomas Fariña Núñez, Alina Glebova, Myriam Peters, Tina Meer, Saskia Opitz, Bianca Lauble, Jolanda Daniuk, Melanie Adler, Junko C. Hübers, Stefan Grau, Daniel Ruess, Catharina Schröter, Patrick Melich, Gina Fürtjes, Stefanie Jünger, Anna-Katharina Meißner, Petra Heiden, Lena Dreher, Niklas von Spreckelsen, Anne Müller, Isabel Franke, Simone Matte, Katarina Drömer, Stefanie Hamacher, Stefanie Stock, Irini Papachristou, Franziska Schwartzkopff, Annika Brüggen, Christoph Barth, Stefanie Quach, Jörg-Christian Tonn, Eva Lehmann-Emele, Karla Steinberger, Christiane Zimmerer, Berend Feddersen, Eva-Maria Trautwein, Stefanie Kolmhuber, Max Spickermann, Theresa Herttrich, Sabine Streitwieser

**Affiliations:** 1 University of Cologne, Faculty of Medicine and University Hospital, Department of Palliative Medicine, Cologne, Germany; 2 University of Cologne, Faculty of Medicine and University Hospital, Department of Neurosurgery, Cologne, Germany; 3 RWTH Aachen University, Medical Faculty, Department of Palliative Medicine, Aachen, Germany; 4 University of Munich (LMU), Faculty of Medicine and LMU University Hospital, Department of Palliative Medicine, Munich, Germany; 5 University of Freiburg, Faculty of Medicine, Department of Palliative Medicine, Freiburg, Germany; 6 University of Cologne, Clinical Trial Centre Cologne (ZKS), Cologne, Germany; 7 University of Cologne, Faculty of Medicine and University Hospital, Institute of Health Economics and Clinical Epidemiology (IGKE), Cologne, Germany; 8 RWTH Aachen University, Medical Faculty, Department of Neurosurgery, Aachen, Germany; 9 Center for Integrated Oncology, (CIO ABCD), Aachen Bonn Cologne Duesseldorf, Germany; 10 University of Freiburg, Faculty of Medicine and University Hospital, Department of Neurosurgery, Freiburg, Germany; 11 Friedrich-Alexander-University Erlangen-Nurnberg, Faculty of Medicine and University Hospital, Department of Neurosurgery, Erlangen, Germany; 12 Northwestern University Feinberg School of Medicine and Malnati Brain Tumor Institute, Northwestern Medicine, Department of Neurological Surgery, Chicago, USA; 13 University of Cologne, Faculty of Medicine, Institute of Medical Statistics and Computational Biology (IMSB), Cologne, Germany; 14 University Medical Center Göttingen, Department of Medical Statistics, Göttingen, Germany; 15 University of Bonn, University Hospital Bonn, Center for Neurology, Department of Neurooncology, Bonn, Germany; 16 University of Bonn, University Hospital Bonn, Department of Palliative Medicine, Bonn, Germany; 17 University Medical Center Göttingen, Department of Palliative Medicine, Göttingen, Germany; 18 Heinrich-Heine-University Düsseldorf, University Hospital Düsseldorf, Interdisciplinary Centre of Palliative Medicine, Düsseldorf, Germany; 19 Heinrich-Heine-University Düsseldorf, University Hospital Düsseldorf, Department of Anaesthesiology, Düsseldorf, Germany; 20 Beta-Klinik Bonn, Center for Neuro-Oncological Neurosurgery, Bonn, Germany; 21 University of Cologne, Faculty of Medicine and University Hospital, Center for Neurosurgery, Department of Stereotaxy and Functional Neurosurgery, Cologne, Germany; 22 University Hospital Munich (LMU), Faculty of Medicine and LMU University Hospital, Department of Neurosurgery, Munich, Germany; 23 University of Bonn, University Hospital Bonn, Department of Neurosurgery, Bonn, Germany; 24 University of Cologne, Faculty of Medicine and University Hospital, Center for Health Services Research Cologne (ZVFK), University of Cologne, Cologne, Germany; 25 Heinrich-Heine-University, Düsseldorf, University Hospital Düsseldorf, Department of Neurosurgery, Düsseldorf, Germany

**Keywords:** Glioblastoma, Randomized Clinical Trial, Palliative care intervention

## Abstract

**Purpose:**

Patients with glioblastoma represent a highly vulnerable cohort as they often experience rapid health deterioration with severe symptom burden including neurological, (neuro)psychological, and psychiatric symptoms. The aim of this sub-analysis of the “Early Palliative Care for Patients with Glioblastoma” (EPCOG) trial was to investigate the specific challenges of conducting a multicenter, randomized, controlled, clinical trial in glioblastoma patients testing a specialized palliative care (PC) intervention.

**Methods:**

We analyzed screening protocols and protocol deviations with respect to number and reasons for non-participation, skipped/delayed visits and attrition using descriptive statistics and content analysis of free-text comments.

**Results:**

In total, 41.5% of 556 screened patients were enrolled. Main reasons for non-participation were lack of interest (25.7%) and low functional status (11.5%). Attrition due to death (57.6%) was higher than due to illness (5.2%) or other reasons (21.2%). Main reasons for visit deviations were structural issues (in > 50% of neurosurgical visits), health status, and patient request. Protocol deviations showed that specialized PC intervention visits were least frequently skipped (4.5%) compared to study-specific outcome assessment (10.1%) and neurosurgical (43.3%) visits. Further, only 11.0% of the specialized PC intervention visits were delayed compared to 22.3% of the outcome assessment and 56.4% of the neurosurgical visits.

**Conclusion:**

In this clinical trial involving glioblastoma patients, a high level of motivation among the study participants could be reached, as reflected by low protocol deviations during the specialized PC intervention and study-specific outcome assessment visits. Reasons for this might be a close guidance as well as a patient and caregiver-oriented communication, e.g., by a personal contact of the PC team in the intervention group, personal outcome assessment visits at patients’ whereabouts, or the inclusion of a study nurse at each site. Considering the high vulnerability of glioblastoma patients is crucial when designing and conducting clinical trials.

**Supplementary information:**

The online version contains supplementary material available at 10.1007/s00520-026-10564-7.

## Introduction

Randomized controlled clinical trials (RCTs) are the gold standard for evidence-based research [1]. However, RCTs can place considerable demands on participants. The enrolment process alone, with extensive information and consent procedures, is usually resource and time-consuming, followed by study-specific actions according to a trial protocol. Nevertheless, patients and relatives are willing and motivated to participate in RCTs as they experience closely monitored disease-related measures, hope for an improvement, and find meaning in sharing their experience which may be of help for future patients [2, 3]. Challenges in conducting RCTs are especially high in particularly vulnerable patient groups such as palliative care (PC) patients and their caregivers, when symptom burden is high and time and energy levels are scarce [4]. These patients experience existential concerns, so that feasibility, benefits, risks, and knowledge gained from the study for clinical practice must be carefully weighed [5–7]. RCTs in PC face challenges with clinicians’ and relatives’ gatekeeping [4, 8, 9], recruitment, compliance, attrition, and missing values and may raise ethical concerns for the patients randomized to the control arm [8–10].

Within the vulnerable group of PC patients, patients with glioblastoma, the most common and most malignant brain tumor, suffer from a rapid deterioration in overall health with a special focus on fast progressive neurological, (neuro)psychological, and psychiatric changes with a high impact on mobility, personality, cognitive abilities, communication, social life, and relationships [11–19]. In addition, many patients with glioblastoma show physical or cognitive limitations already before diagnosis and suffer from a fast deterioration, even when showing a high performance score [20, 21], which may complicate inclusion and continued participation in a RCT and lead to a high attrition rate. Therefore, conducting RCTs in this vulnerable patient population, which may be considered as an exemplary disease of brain tumor patients, raises specific challenges that we could learn from for the planning of future RCTs including these patients. The aim of this study was, therefore, to investigate these particular challenges in conducting a RCT based on the EPCOG trial (Early PC for patients with glioblastoma [22]).

## Methods

### Study procedure

The multicenter, randomized, confirmatory, phase III, rater-blinded, parallel-group clinical EPCOG trial (EPCOG) was conducted at the Departments of Palliative Medicine and the Departments of Neurosurgery of the University Hospitals of Aachen, Bonn (here, additionally the Department of Neuro-oncology), Cologne, Düsseldorf, Freiburg, and Munich, in accordance with the Declaration of Helsinki [23], approved by the local ethics committees (Cologne, #19-1024_7), and registered in the German Clinical Trials Register (#DRKS00016066).

The study protocol as well as the main results of the EPCOG study was published elsewhere [16, 24].

Patients were screened for eligibility at the Departments of Neurosurgery (Bonn, also the Department of Neuro-oncology). Written informed consent was obtained within 4 weeks of initial or recurrent diagnosis from patients and their caregivers,^1^ who could also participate in the trial. Screening procedures were documented in each center including free-text comments in case of screening failures. Enrolled patients were randomized to either the control or intervention group following a baseline assessment. In the intervention group, patients received a specialized PC intervention within the first 12 months of the study, consisting of visits by a specialized PC physician and a PC social worker in person (every 3 months (*n* = 4)) and via telephone contacts (on a monthly basis in between (*n* = 8)). The intervention consisted of collection of issues and implementation of measures related to pain and symptom management, psychosocial and spiritual support, assistance in treatment, decisions, and help in care planning [22]. In both groups, patients underwent standard neurosurgical/neuro-oncological follow-ups (neurosurgical visits) every 3 months after diagnosis, which were optimized by the use of the Functional Assessment of Cancer Therapy-Brain (FACT-Br) as defined in the study protocol [22]. Study-specific outcome assessment was conducted every 3 months by a researcher blinded for the intervention. Outcome assessments were carried out at patients’ whereabouts within the first 12 months and via telephone in the 12 months of follow-up. For more details concerning the study design, please also see Annex I. Study participants were asked not to speak about their randomization result [22].

The primary objective of the overall study was to determine patients’ quality of life after 6 months when receiving specialized PC compared to the control group. Quality of life was assessed by the trial outcome index (TOI) encompassing physical, functional, and brain-specific factors of the FACT-Br following Temel et al. [25, 26]. Secondary objectives were PC needs measured by the Integrated Palliative Outcome Scale (IPOS [27, 28]), anxiety and depression measured by the Hospital Anxiety and Depression Scale (HADS) [29], and patients’ cognitive abilities measured by the Montreal Cognitive Assessment (MoCA) [30–32]. To assess caregiver burden, the 12-question short form of the Zarit Burden Interview (ZBI-12) [33–36] was used. Healthcare use was recorded using a study-specific questionnaire. Any collected data as well as study participants’ inclusion, enrollment, randomization, all visit dates, and individual end of study were entered into a good clinical practice-compatible electronic case-reporting form database (OmniComm TrialMaster).

### Consideration of specific study population

When designing the EPCOG trial, we considered the specific challenges of the vulnerable patient population of patients with glioblastoma [9, 37, 38], expecting a low recruitment rate of one third of all screened patients and a high attrition rate of 40% [10, 11, 39]. A correspondingly high number of patients were scheduled for screening and recruitment in a sufficient number of study centers. Patients with first and recurrent glioblastoma diagnoses could be included, not only to allow for a comparison of both groups but also to keep inclusion criteria as broad as possible within the chosen study population [9, 10]. For detailed inclusion and exclusion criteria, please see Table 1.

**Table 1:** Key inclusion and exclusion criteria [22]

	Patients	Caregivers
Inclusion criteria	• Patients with newly diagnosed GB (histologically confirmed by biopsy or resection) **within 4 weeks after diagnosis** **or** • Patients with recurrent GB **within 4 weeks after diagnosis** of recurrence (confirmed according to RANO criteria and/or radiological deterioration leading to a change in oncological treatment as indicated by the investigator)	• Caregiving persons (relatives or other closely related persons) of special importance for the patients, i.e., they live with them or have face to face contact with them at least twice a week **Note:** Patients can also be included if no such caregiver exists
	**and**	
	• ECOG 0–2* • Age ≥ 18 years • Ability to understand, read and respond to the German language • Ability to give informed consent	
Exclusion criteria	• Unwillingness to abide by the protocol • Being legally incapacitated • On-going drug abuse or alcohol abuse or a psychiatric condition that, in the opinion of the investigator makes the patient or caregiver unsuitable for study participation • Any kind of dependency on the investigator or employed by the sponsor or investigator • Held in an institution by legal or official order	

* GB * glioblastoma; *Eastern Cooperative Oncology Group [ECOG] performance status: Grade 0: fully active, able to carry on all pre-disease performance without restriction; Grade 1: restricted in physically strenuous activity but ambulatory and able to carry out work of a light or sedentary nature, e.g., light house work, office work; Grade 2: ambulatory and capable of all selfcare but unable to carry out any work activities; up and about more than 50% of waking hours; Grade 3: capable of only limited selfcare; confined to bed or chair more than 50% of waking hours; Grade 4: completely disabled; cannot carry on any selfcare; totally confined to bed or chair; Grade 5: dead

The recruitment period was set to 24 months, with one study nurse per site assuring a smooth recruitment process. To reduce missing values due to anticipated patients’ health deterioration, joint (patient and caregiver) and proxy (caregiver only) assessments were allowed a priori except for the MoCA (only self-assessment). Additionally, missing values should be reduced by mainly face-to-face outcome assessments with consistent, familiar researchers guiding through the questionnaires [37]. To keep the data collection as short as possible, the information on health care use was completed by the researchers in advance via patients’ records for the respective hospital. A compliance form was completed by the researcher documenting the type of assessment (self, joint, or proxy), blinding, and reasons if a visit or completion of a single questionnaire was not possible.

### Study specific analysis of protocol violations

Unlike protocol violations, protocol deviations do not have significant consequences for the trial (although being in non-conformance of the trial protocol) [40]. In the EPCOG trial, protocol deviations provided the basis for specific information on challenges that influenced the overall study process. Paper-based screening lists were manually scanned for reasons of screening failure and frequency of non-inclusion. End-of-study forms were used to categorize and count attrition according to the MORECare criteria [41]. A bypass list consisting of responded free-text fields provided by the system whenever a specific answer was required but did not match the configuration was generated by the study database TrialMaster. It was analyzed for frequency and reasons for skipped or delayed visits using Microsoft Excel 2019.

Compliance forms were used to extract quantitative and qualitative characteristics of study visits including type of assessment, frequency of, and reasons for skipped questionnaires and data collector’s unblinding.

Free-text comments from compliance forms and bypasses were qualitatively analyzed using inductive qualitative content analysis [42], creating main and subcategories which were interprofessionally discussed in an iterative process until consensus relating to the hierarchy and content of the categories was reached. Afterwards, the frequency of each category was counted. SPSS Statistics 29 (IBM Corp., Armonk, NY, USA) was used for descriptive statistics (i.e., absolute and relative frequencies) of the compliance forms. 

## Results

### Screening, non-participation, and attrition

**Fig. 1:**
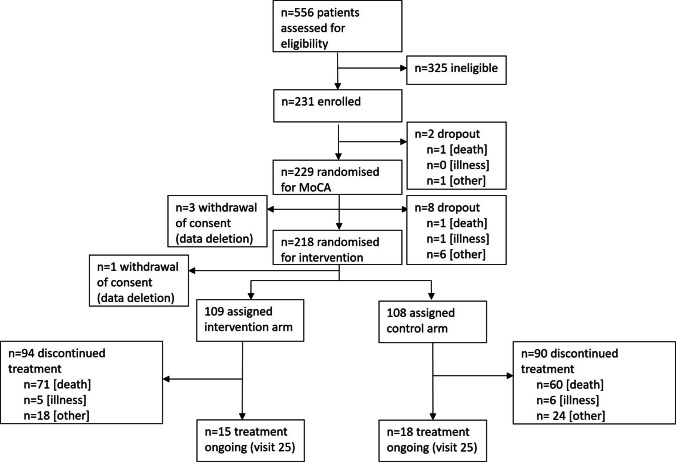
Screening, non-participation and attrition of patients in the overall study, from screening to regular end of study (visit 25)

In total, the inclusion rate was 41.5% for patients and 35.0% for caregivers (*n* = 556). Reasons for non-participation (screening failure) were related to patients in 85.8% of the cases, i.e., lacking interest (*n* = 143; 25.7%), low functional status (ECOG > 2) (*n* = 64; 11.5%), and strain (*n* = 18; 3.2%), or cognitive impairment and language barriers, e.g., aphasia (*n* = 16; 2.9%). In 14.2% of the cases, structural and organizational reasons led to non-participation; here, main reasons were a neurosurgical treatment in a department other than the recruiting department (*n* = 27; 4.9%) and the impossibility to meet patients (*n* = 14; 2.5%). After study inclusion, 133 patients dropped out of the study due to death (57.6%), 12 patients dropped out due to illness (5.2%), nine patients dropped out for other reasons (21.2%), four patients requested data deletion before the regular end of the study (1.7%), and 33 patients finalized the study per protocol (14.3%) (see Fig. 1).

### Skipped or delayed visits

**Fig. 2:**
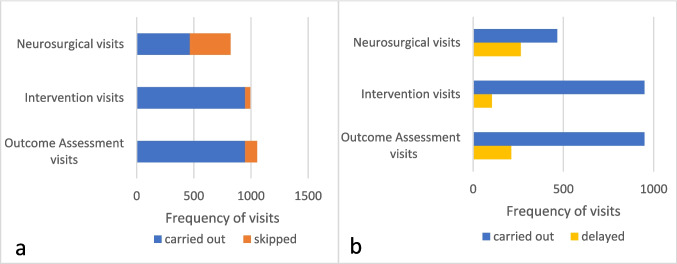
Frequency of carried out, skipped and delayed visits. **a** Ratio of skipped visits was 356/822 (43.0%) for neurosurgery visits, 45/993 (4.5%) for specialized palliative care intervention visits, and 106/1054 (10.1%) for study-specific outcome assessment visits. **b** Ratio of delayed visits was 263/466 (56.4%) for neurosurgical visits, 104/948 (10.9%) for specialized PC intervention visits and 201/946 (21.2%) for outcome assessment visits

**Fig. 3:**
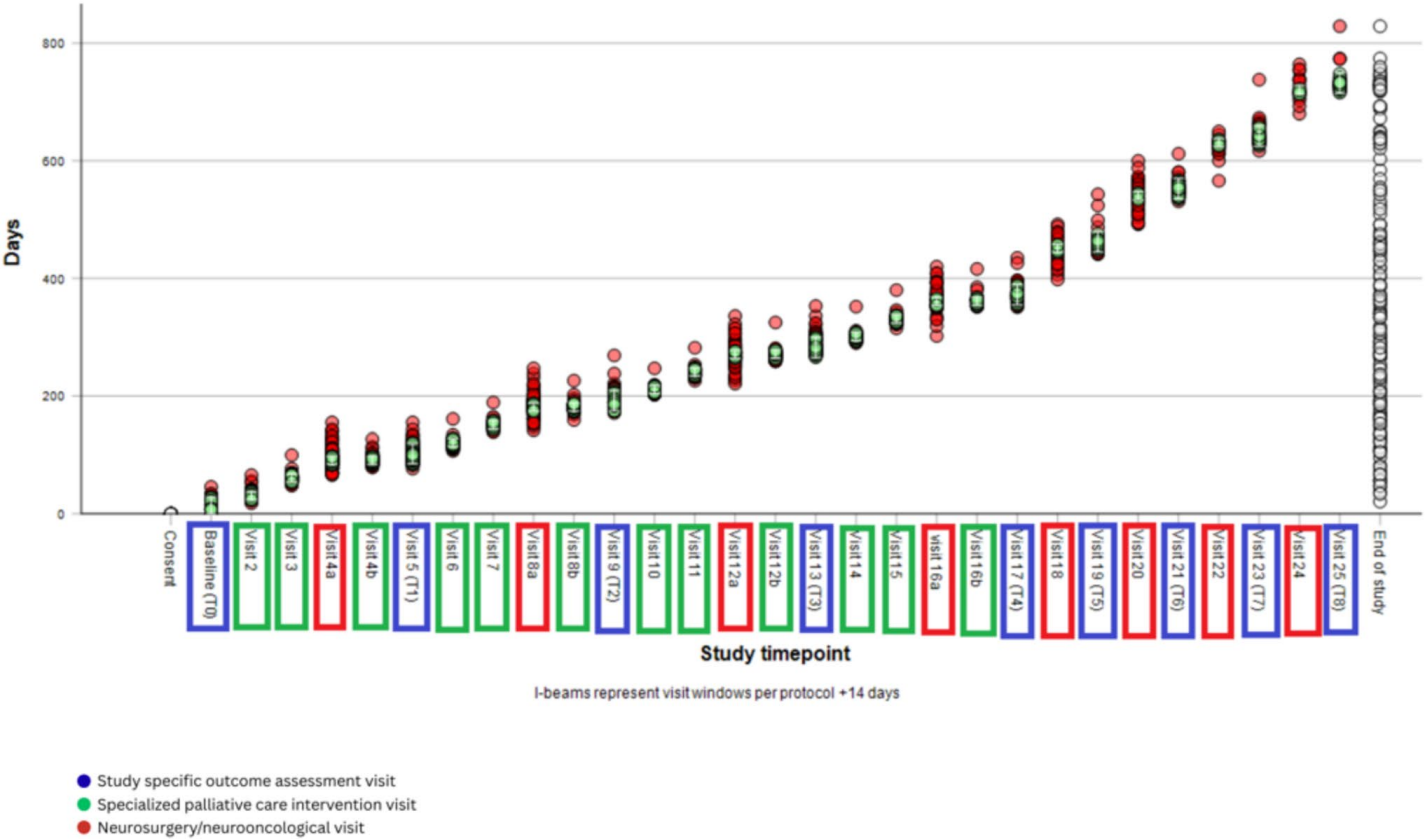
Timepoint accuracy of the total visits for the entire study population. Green features represent accumulated punctual visits; red features represent accumulated non-punctual visits. Neurosurgery visits (red): 4a, 8a, 12a, 16a, 18, 20, 22, 24; palliative care intervention visits (green): 2, 3, 4b, 6, 7, 8b, 10, 11, 12b, 14, 15, 16b; outcome assessment visits (blue): baseline, 5, 9, 13, 17, 19, 21, 23, 25

Ratio of skipped and delayed visits is shown in Fig. 2. The timepoint accuracy of all visits for the entire study population is shown in Fig. 3.

In total, 2656 bypasses of all study participants with free-text specifications were analyzed. Reasons for skipped and delayed visits could be categorized into four main categories for all types of visits, i.e., *structural issues*, *health condition, patient preference*, and *other*, and 18 subcategories in total*.* However, main and subcategories showed different emphases for different types of visits (see Table 2).

**Table 2:**
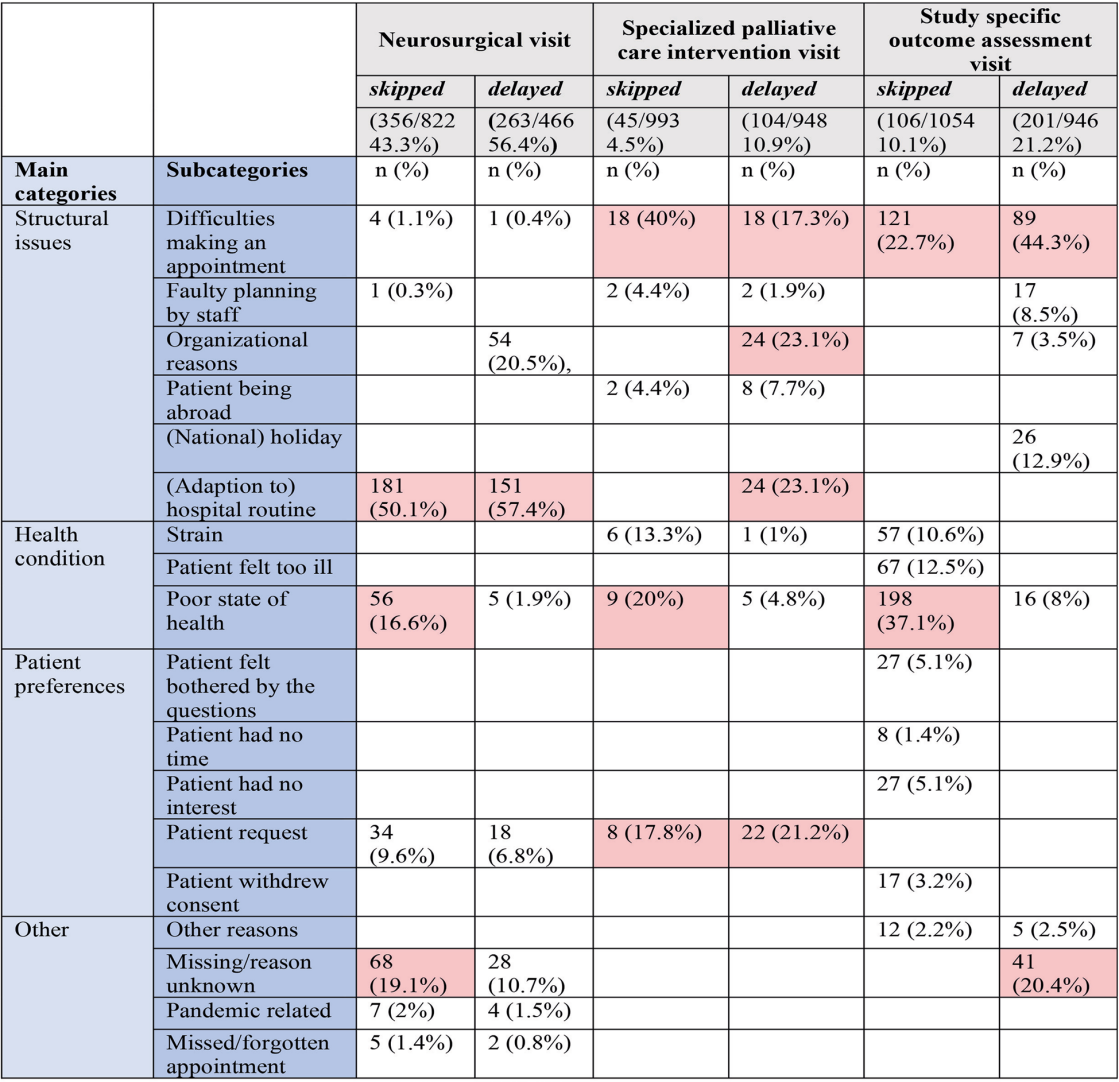
Main and subcategories of skipped and delayed neurosurgical, specialized palliative care intervention and study specific outcome assessment visits; multiple answers were possible (frequency > 15% highlighted in red)

#### Neurosurgical/neuro-oncological visits

Neurosurgical/neuro-oncological visits were skipped (43.3%) or delayed (56.4%) most frequently of all study visits. The main category *structural issues* with the subcategory *(adaption to) hospital routine* was mostly represented for skipped or delayed neurosurgical visits; i.e., they were no longer medically necessary, either because of a change in therapy (further treatment in another department or clinic), a time shift in appointments due to the course of the disease or treatment (reoperation, radio-therapy), delayed (internal/external) MRI appointments, or appointments made by the neurosurgeon that were not in accordance with the trial protocol. We identified one subcategory (*poor health*) within the main category *health condition* and one subcategory (*patient request*) within the main category *patient preferences*, e.g., visit schedule outside the study-specific time like lack of an accompanying person for a study visit.

#### Specialized palliative care intervention visits

Specialized PC intervention visits were rarely skipped (4.5%) or delayed (11%). Here, the main reasons also fall into the main category *structural issues*, and herein specifically into the subcategory *difficulty in making an appointment*; i.e., no time for an appointment could be found in agreement with the patient and/or caregiver, or they were not available. *Organizational reasons* and *hospital routine* played a significant role. This was due to the study design, which scheduled personal specialized PC intervention visits on the same day as routine neurosurgical visits to minimize the burden on study participants by requiring only one hospital visit. Neurosurgical visits that did not fall within the time frame of the study design therefore affected the timeliness of specialized PC intervention visits. *Patient request* within the main category of *patient preference* played a considerable role in changing the timing of the specialized PC visit, for example, due to the absence or presence of urgent PC needs of the patient or caregiver.

#### Study specific outcome assessment visits

The compliance form detailed the reasons for skipped or delayed outcome assessment visits, resulting in more specific subcategories compared to neurosurgical visits and specialized PC intervention visits, but the main categories were the same (*structural issues*, *health condition*, *patient preference*, *other reasons*). The main reasons for skipping or rescheduling an outcome assessment visit were clearly *difficulties in making an appointment* and *poor state of health*. However, while the latter reason mostly led to a complete cancellation of the visit, in the overwhelming majority of cases, the difficulty in getting an appointment enabled the outcome assessment visit to take place, even if it was delayed. Free-text comments about the MoCA test reflected the cognitive abilities required, which declined over time; thus in the subcategory *poor state of health* (*n* = 111; 48.9%), aphasia (*n* = 18; 7.9%) and lack of concentration (*n* = 10; 4.4%) were reported.

### Compliance

#### Assessment type

**Fig. 4:**
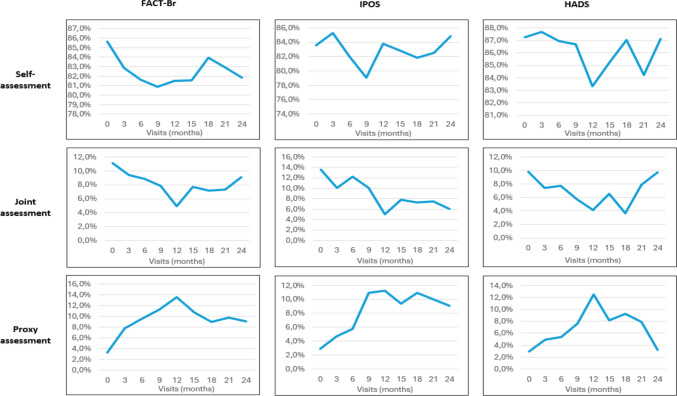
Trajectories of self-, joint, and proxy assessment for Fact-Br, IPOS, and HADS. The figure depicts proportions of self-, joint, and proxy assessment by visit and outcome for FACT-Br (quality of life), IPOS (palliative care issues), and HADS (psychological strain) during the course of the study (9 outcome assessments in 24 months)

A mean of 84.1% of all completed questionnaires during outcome assessment visits was self-assessed. A mean of 8.8% was completed as joint-assessment, and a mean of 7.1% was completed as proxy assessment (for details for each questionnaire, see Fig. 4). In the case of proxy assessment, reasons for not completing the questionnaire(s) were as follows: *no proxy was available* (*n* = 19; 13.4%), *proxy did not feel competent to answer the questions on behalf of the patient* (*n* = 28; 19.7%), *proxy* *felt too strained* (*n* = 49; 34.5.3%), *had no time* (*n* = 26; 18.3.7%), *no interest* (*n* = 11; 7.7%), or *proxy* *felt bothered by the questions* (*n* = 13; 9.9%). For trajectories for each questionnaire and assessment type, see Fig. 4.

#### Completion of questionnaires

The MoCA questionnaire was most frequently not completed (*n *= 251; 26%), followed by the HADS (*n* = 88; 9.3%), IPOS (*n *= 47; 5%), and FACT-Br (*n* = 10, 1.1%). The health economic questionnaire was not completed in 140 cases (12%). Reasons for non-completion of single questions of the questionnaire(s) were as follows: *patients not able to answer in this phrasing* (reality too complex) (*n* = 43; 14%), *patient declined* *to answer* (*n* = 35; 11%), and *proxies* *did not feel competent to answer on behalf of the patient* (*n* = 38; 12%).

Caregiver burden, collected with the ZBI-12, was not answered in 24.1%, most frequently due to *no possibility to meet the caregiver* (*n* = 127; 51%) and to *declining answering the questionnaire* (*n* = 148; 59.4%) including *caregiver burden* (*n* = 58; 39.2%) and other reasons (*n* = 93, 62.8%). Multiple answers were allowed.

#### Researchers’ unblinding

The researcher was unblinded in 52 cases (24%): 12 (5%) in the control and 40 (18%) in the intervention arm. Reasons for unblinding were most frequently mentioning names of or contacts to PC intervention staff (*n* = 16; 30.8%) and concerns about the study design, e.g., the end of the intervention or the study staff’s responsibilities (*n* = 9; 17.3%).

## Discussion

The EPCOG trial was the first RCT to investigate the impact of early specialized PC on this vulnerable patient cohort and their caregivers. Albeit its complex design, we achieved high recruitment and compliance rates.

This RCT was feasible with a sufficient number of patients for two main reasons. First, we took essential precautions to conduct the trial (e.g., considering the study participants’ limited capacity, the expected high screening failure and attrition rate, the use of constant, familiar staff for outcome assessment, and support by a study nurse for recruitment processes). Additionally, communication was maintained as much as possible on a personal level during the 12 months following enrollment, by the researchers, the neurosurgeons, and the specialized PC intervention team, respectively. Second, we implemented an intervention that was likely to be considered appropriate by both the study participants and the investigators.

The inclusion of a study nurse at each study site, who was not involved in patient care, provided an objective review of study eligibility. This ensured that gatekeeping from health care professionals during the recruitment process was minimized, which is a major challenge in PC trials, especially when using a RCT design [43]. The study nurses also helped to facilitate interdepartmental study processes and were important contacts not only for study staff but also for study participants. This is consistent with previous findings that compassionate research assistants (e.g., study nurses) are key facilitators for conducting clinical trials, especially during recruitment processes [10].

Caregiver involvement in clinical trials minimizes gatekeeping by caregivers, and caregivers are more likely to participate in a trial when the patient is participating [9, 43]. These findings support our study design, which involved both patients and caregivers, probably positively impacting on recruitment, blinding, and attrition. This may also be an important consideration for studies involving patients with other types of tumors or other neurological diseases, which impact cognition. However, since patients with glioblastoma experience both physical and neurological symptoms, it is even more important for this patient group to address both challenges in the study design (e.g., conducting outcome assessments on a personal basis at home, involving caregivers for higher overall participation, and avoiding missing data in case the patient is no longer able to complete the questionnaire or to communicate).

Study participants’ interest in the study was reflected in a recruitment process that followed exactly the study protocol. In particular, according to the relatively low number of missed or delayed specialized PC intervention and outcome assessment visits and the overwhelming degree of self-assessments in this particularly vulnerable patient population, we found that patients were highly motivated to attend specialized PC intervention and outcome assessment visits. This is also reflected in the fact that attrition due to other reasons and illness was 2.2 times lower than attrition due to death. This suggests that patients found it worthwhile to participate in the trial, and therefore the predominant reason for attrition was unavoidable death rather than other reasons. In contrast, adherence to neurosurgical visits was worse, probably reflecting the clinical reality of patients being referred from neurosurgery to neuro-oncological and other in-house departments for further treatment. Also, the process of being informed about the results by the radiologist concerning the MRI beforehand may lead to less motivation of making an appointment for a neurosurgical visit. Additionally, deviations from regular neurosurgical appointments might in some instances have been related to prior unscheduled patient revisits due to clinical deterioration. However, a certain prioritization of the specialized PC intervention over neurosurgical visits—at least within the intervention group—may have led to less neurosurgical visits.

With careful planning, we achieved an inclusion rate of 41.5% which is within the range of other PC studies [9, 37, 44–46]. Reasons for non-participation were similar to other PC studies [8, 47]. Overall attrition due to death was comparable to a study with similar time frame including patients with malignant glioma and glioblastoma [48], but substantially higher than in specialized PC intervention trials including oncology patients with systemic solid malignancies [49–51], highlighting the vulnerability and the related challenges of glioblastoma patients. It is even more important to emphasize that this interventional trial was conducted with relatively few disruptions despite the Covid-19 pandemic, in contrast to others [52]. Study participants were open to alternative ways of meeting (i.e., by telephone to reduce risk of infection transmission), with the effect that study visits were not significantly skipped or delayed.

Unblinding occurred only in about a quarter of patients. This is much less than expected given the stressful situation that patients and caregivers are in, as well as patients’ cognitive impairment and the challenge to remember with whom (study nurse, PC physician, social worker, and researcher) they could talk openly about the study intervention or unmet needs in the control group. This again reflects the motivation and identification of the study participants with the trial.

## Limitations

The trial focused on neurosurgical visits for glioblastoma-specific follow-up. However, it would have been helpful to include more neuro-oncology and radiotherapy departments as study partners to achieve a higher follow-up rate for tumor-specific treatment. However, our decision not to do so was based on a desire not to increase the complexity of the study any further.

This study reports on challenges in recruiting patients with glioblastoma for palliative care in a RCT setting. This does not reflect the practice in neurology or neurosurgery departments of enrolling patients in palliative care, as study nurses are not typically hired for this purpose. Optimizing methods of providing palliative care for this patient group would be a promising area for future research.

## Conclusion

RCTs in vulnerable PC study populations are feasible, but require rigorous planning, adequate staffing, and a targeted study protocol, which is time-consuming and costly. However, it is the only way to achieve meaningful evidence. The inclusion of vulnerable PC patients in complex trials can only be justified if reliable planning has taken place. This study contributes to this understanding.

## Supplementary information

Below is the link to the electronic supplementary material.

ESM 1 (PDF 178 KB)

## Data Availability

Data are available upon reasonable request from the corresponding author.

## Footnotes

1 The term “caregiver” is most widely used to refer to non-professionals assisting in the care of persons receiving (palliative) care. Since not all of them were family members, the term “family caregiver” did not fit our purposes and the term “informal caregiver” has yet been discussed to be inappropriate (see [53]). That is why we chose the unspecific term “caregiver” for the next of kin of the patients caring for them.
