# Supplementary material for: Conducting a randomized controlled clinical trial on palliative care in patients with glioblastoma – what are the challenges?
Source: Support Care Cancer. 2026 Apr 27;34(5):468. doi: 10.1007/s00520-026-10564-7 (PMC13121353; doi:10.1007/s00520-026-10564-7)
Supplement: Supplementary file 1 — (PDF 178 KB) [file 520_2026_10564_MOESM1_ESM.pdf]

Annex I

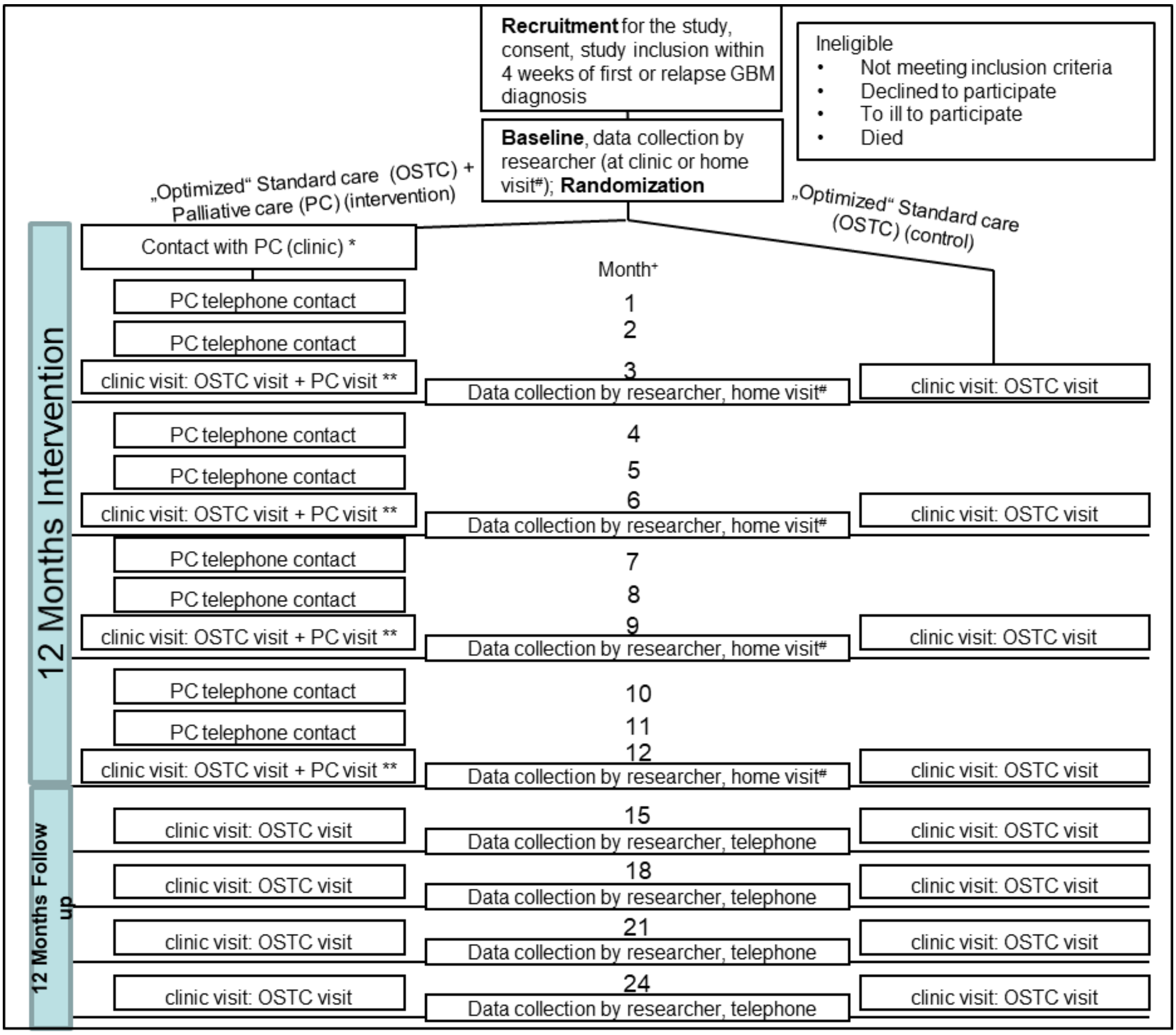

Golla, H.; Nettekoven, C.; Bausewein, C.; Tonn, J.-C.; Thon, N.; Feddersen, B.; Schnell, O.; Böhlke, C.; Becker, G.; Rolke, R.; et al. Effect of early palliative care for patients with glioblastoma (EPCOG): a randomised phase III clinical trial protocol. *BMJ Open* 2020, *10*, e034378, doi:10.1136/bmjopen-2019-034378.

Detailed study design

The assessment researcher visited the patient for data collection at home or his/her whereabouts. All visits were allowed to be scheduled within a time frame of ±1 week except for the assessment visits for data collection at patient’s home/whereabouts every 3 months. These assessment visits had to be scheduled after the respective clinic visit (and PC visit, intervention group, only) within a time frame of +2 weeks. *After randomization and before first PC contact by telephone (no later than 4 weeks after study inclusion): first contact of PC physician and PC social worker (PC intervention team) with patients/caregivers to introduce themselves (not yet an intervention visit, but solely served the purpose of getting acquainted with each other before the first PC intervention contact by telephone). **If patient was too ill for hospital visit, telephone contact with the PC intervention team instead. OSTC: optimized standard care; the optimization of the standard care was achieved by regular assessments of the patients’ quality of life at each regular hospital visit using the Functional Assessment of Cancer – Brain (FACT-Br) questionnaire; PC: Palliative Care.
